# Role of Exosomes in Salivary Gland Tumors and Technological Advances in Their Assessment

**DOI:** 10.3390/cancers16193298

**Published:** 2024-09-27

**Authors:** Artur Nieszporek, Małgorzata Wierzbicka, Natalia Labedz, Weronika Zajac, Joanna Cybinska, Patrycja Gazinska

**Affiliations:** 1 Biobank Research Group, Łukasiewicz Research Network–PORT Polish Centre for Technology Development, Stablowicka Street 147, 54-066 Wroclaw, Poland; 2 Institute of Human Genetics Polish Academy of Sciences, Strzeszynska 32, 60-479 Poznan, Poland; 3 Department of Otolaryngology, Regional Specialist Hospital Wroclaw, Research & Development Centre, Kamienskiego Street 73a, 51-124 Wroclaw, Poland; 4 Faulty of Chemistry, University of Wroclaw, Joliot-Curie 14, 50-383 Wroclaw, Poland; 5 Materials Science and Engineering Center, Łukasiewicz Research Network–PORT Polish Centre for Technology Development, Stablowicka Street 147, 54-066 Wroclaw, Poland

**Keywords:** saliva-derived exosomes, salivary gland tumors, liquid biopsy, exosomes, tumor microenvironment, saliva, biosensor

## Abstract

**Simple Summary:**

Salivary gland tumors (SGTs) are rare and complex, making them difficult to diagnose and treat. This work focuses on the role of exosomes, extracellular vesicles secreted by almost all cell types, in the development and progression of SGT. Exosomes are crucial in cell-to-cell communication and play significant roles in tumor biology, including modulating the tumor environment, aiding metastasis, and affecting immune responses. A better understanding of exosome biology can lead to their use as biomarkers for diagnosis and prognosis, as well as targets for treatment, potentially transforming SGT management and improving patient outcomes. Exosome-based liquid biopsies could offer non-invasive, real-time diagnostics and enhance patient care through precision medicine. This review explores the advancements in salivary exosome analysis, highlighting its potential for non-invasive cancer detection and the development of innovative diagnostic techniques.

**Abstract:**

**Backgroud:** Salivary gland tumors (SGTs) are rare and diverse neoplasms, presenting significant challenges in diagnosis and management due to their rarity and complexity. Exosomes, lipid bilayer vesicles secreted by almost all cell types and present in all body fluids, have emerged as crucial intercellular communication agents. They play multifaceted roles in tumor biology, including modulating the tumor microenvironment, promoting metastasis, and influencing immune responses. **Results**: This review focuses on the role of exosomes in SGT, hypothesizing that novel diagnostic and therapeutic approaches can be developed by exploring the mechanisms through which exosomes influence tumor occurrence and progression. By understanding these mechanisms, we can leverage exosomes as diagnostic and prognostic biomarkers, and target them for therapeutic interventions. The exploration of exosome-mediated pathways contributing to tumor progression and metastasis could lead to more effective treatments, transforming the management of SGT and improving patient outcomes. Ongoing research aims to elucidate the specific cargo and signaling pathways involved in exosome-mediated tumorigenesis and to develop standardized techniques for exosome-based liquid biopsies in clinical settings. **Conclusions:** Exosome-based liquid biopsies have shown promise as non-invasive, real-time systemic profiling tools for tumor diagnostics and prognosis, offering significant potential for enhancing patient care through precision and personalized medicine. Methods like fluorescence, electrochemical, colorimetric, and surface plasmon resonance (SPR) biosensors, combined with artificial intelligence, improve exosome analysis, providing rapid, precise, and clinically valid cancer diagnostics for difficult-to-diagnose cancers.

## 1. Introduction

Salivary gland tumors (SGTs) are rare, diverse neoplasms with an incidence of 2.5–3.0 per 100,000 per year in Western countries, with approximately 80% being benign. Malignant types comprise less than 0.5% of all malignancies and up to 5% of head and neck cancers. The World Health Organization (WHO) lists nearly 40 types of epithelial tumors, many of which are extremely rare. Their rarity and diversity make diagnosis and management challenging and often require specialist centers [1]. Salivary gland malignancies (SGMs) are characterized by unpredictable growth expansion, considerable perineural invasion, risk of regional lymph node invasion, and a high risk of distant metastasis, which eventually leads to low survival outcomes [2,3].

Complete excision is recommended for patients with benign tumors. Long-term follow-up is recommended after intraoperative tumor spillage, with multidisciplinary team discussions on adjuvant radiotherapy (RT) [4]. Surgery and adjuvant RT is recommended for all malignant submandibular tumors, high-grade and large tumors, or when resection margins are incomplete, except for small, low-grade, and completely excised SGM.

However, there have been recent methodological and technological advances, such as simultaneous profiling of the genome, epigenome, transcriptome, proteome, and other emerging omics modalities, that have facilitated a deeper understanding of biological mechanisms and genotype-to-phenotype relationships [5]. New omics technologies are emerging for the discovery of novel biomarkers and therapeutic targets, contributing significantly to the progress of precision and personalized medicine [6]. Additionally, liquid biopsies have been established as powerful research tools, enabling the detection and quantification of biomarkers by analyzing non-solid biological samples. Initially used to detect circulating tumor cells in breast cancer [7], liquid biopsies now encompass various fluids, including blood, saliva, urine, and cerebrospinal fluid [8]. Exosome-based liquid biopsies are a promising method for disease diagnosis and prognosis, as well as a technique for swift identification and continuous tracking of cancer treatment efficacy [9]. These biopsies are non-invasive, easy to collect, and provide real-time systemic profiles and spatiotemporal information [10].

Exosomes are lipid bilayer membrane vesicles of endosomal origin with a diameter of 40–150 nm [11], enriched with tetraspanin membrane proteins, such as CD9, CD37, CD63, CD81, and CD82. Additionally, proteins such as TSG-101 and Alix from the exosome sorting complex required for transport (ESCRT) are commonly used as markers [12,13].

Exosomes are secreted by almost every cell type and are ubiquitously present in all body fluids [14]. They are defined as a subset of intraluminal vesicles (ILVs) secreted following the fusion of multivesicular bodies (MVBs) with the plasma membrane. Exosomes are extracellular vesicles (EVs) that mirror the physiological states of tumors, which change over time and are affected by metabolic processes, the tumor microenvironment (TME), and drug selection pressures [15]. These vesicles, specifically the exosome subset, similarly reflect the dynamic nature of tumor physiology and are shaped by metabolic factors, the surrounding environment, and the pressures exerted by drug treatments [16]. Within the past decade, EVs have emerged as critical mediators of intercellular communication, transmitting biological signals between cells to regulate a diverse range of biological processes [17]. Exosomes, which contain bioactive cargo, serve as biomarkers for cancer diagnosis and therapy. Exosomes from tumor and stromal cells mediate drug resistance by regulating drug efflux and metabolism, pro-survival signaling, epithelial–mesenchymal transition, stem-like properties, and TME remodeling [18].

The roles of exosomes in SGTs and the mechanisms underlying their effects remain unclear, presenting a significant gap in our understanding of tumor biology. Specific exosomal components that influence the progression and metastasis of SGTs have not yet been identified, making it challenging to develop targeted therapies.

In this review, we aim to comprehensively explore the multifaceted roles of saliva and saliva-derived exosomes (SEs) in terms of their composition, analytical potential, and implications for studying SGTs. By addressing these objectives, we sought to provide a thorough understanding of the diagnostic, prognostic, and therapeutic potential of saliva and SEs, particularly, with regard to SGM and oral cancers.

SGMs are rare neoplasms, accounting for less than 1% to 5% of all head and neck cancers [19,20]. Due to their rarity, there is a lack of clinical trial data and formal, evidence-based treatment guidelines, which presents challenges to research efforts. Limited clinical data and biological samples hinder the advancement of understanding, the development of effective therapies, and the ability to conduct extensive research on SGMs [21].

## 2. Composition and Analytical Potential for Studying Exosomes: Advances in Saliva Diagnostics and Exosome Analysis for Non-Invasive Cancer Detection

Saliva is a mixture of oral fluids consisting of more than 99% water [22]. The composition of saliva is not constant and is related to circadian rhythms [23]. Saliva is rich in minerals and electrolytes, such as calcium, zinc, magnesium, sodium, potassium, bicarbonate, and phosphate. It also contains hormones such as adrenomedullin, enzymes such as α-amylase, immunoglobulins, cytokines, antimicrobial peptides, glycoproteins such as lactoferrin and mucins, and oral tissue repair factors such as epidermal growth factor and histatins. Additionally, it includes nitrogenous products, such as urea and ammonia [24,25]. Therefore, analyzing saliva and examining its metabolic profile can serve as valuable tools for diagnosing and predicting non-communicable diseases, including those related and unrelated to oral health [26].

SEs offer significant advantages over exosomes from other sources, owing to their accessibility and ease of collection. Saliva collection is straightforward, non-invasive, cost-effective, does not clot, and is easier to handle [27]. Consequently, SEs have been investigated as alternatives to whole saliva, which contains contaminants and has elevated amylase levels [28]. Challenges with SEs include variability in the salivary flow rate and composition influenced by factors such as sampling methods, diet, age, and health conditions [29]. This variability complicates the standardization of protocols for isolating SEs. Stabilization is also an issue because whole saliva contains a unique mixture of enzymes that are resilient to protease inhibition. Studies have shown difficulties in effectively using protease and RNase inhibitors to prevent protein and RNA degradation [30]. In addition, precise techniques for quantifying the molecular content of SEs are lacking, which hampers their use in targeted therapies.

**Figure 1:**
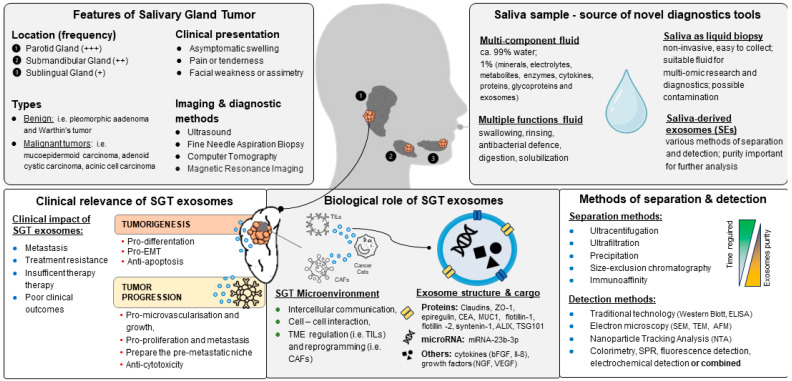
Overview of salivary gland tumor (SGT) exosomes: biological role, clinical relevance, and methods of separation and detection (frequency explanation: +++ (most common), ++ (common), + (rare).

Despite these challenges, understanding the components and functions of SEs is crucial to advancing disease diagnostics and therapies. Liquid biopsy is a minimally invasive technique that allows for the collection of clinical samples as diagnostic tools for early cancer detection, providing information on tumor progression and treatment response [31]. Saliva diagnostics could provide a less invasive and more consistent approach to detecting biomarkers [32]. Thus, it is crucial to develop diagnostic tools that enable the rapid and precise detection of biomarkers such as EVs in salivary gland pathology (Figure 1). To improve the reliability of saliva studies for biomarker analysis, clear protocols must be established. Researchers should specify whether the saliva is whole or from a specific gland and detail the collection methods and any stimuli used. Standardizing or documenting pre-collection conditions, such as food and drink intake, is also critical, as these factors significantly impact saliva composition. Adopting these standards will reduce variability and enhance the comparability of findings across studies [33].

Current methods for exosome isolation include ultracentrifugation, chemical precipitation, ultrafiltration, and exclusion chromatography (Table 1) [34,35,36,37,38,39,40,41,42,43,44,45,46,47,48,49,50,51,52,53,54]. While each technique has advantages, they are also time-consuming, can produce low yields, require many samples, are based on complex protocols, and result in low-purity materials [55,56]. Additionally, using various methods for exosome isolation can lead to inconsistencies in exosome purity, size, zeta potential, and concentration [57,58]. To yield reliable and reproducible results, a single exosome purification method must be consistently applied throughout the experiments, which is especially important when developing exosome-based analytical methods as potential diagnostic tools. For instance, Zlotogorsk et al. employed two exosome isolation methods, chemical and physical (ultracentrifugation), to ensure the robustness of the findings. Coupling both methods confirmed that differences in exosome concentration and size between patients with oral cancer (OC) and healthy individuals were not dependent on a single technique, thus increasing the reliability of the results. Chemical precipitation offers a faster, clinically suitable approach, while ultracentrifugation provides a high-purity standard ideal for research. This dual-method approach enables a comprehensive understanding of exosome characteristics, supporting their potential use in OC diagnostics and therapy [59]. Although differences in exosome characteristics hold potential for diagnostic applications, achieving consistent testing results warrants a standardized methodology. Therefore, the choice of isolation method should be guided by the specific research objectives and requirements for exosomes, carefully weighing the advantages and disadvantages of each approach. Consistency in methodology is pivotal to ensuring reliable and reproducible outcomes in both research and clinical applications.

Nevertheless, the aforementioned factors present a considerable obstacle to further research. From the perspective of point-of-care and rapid, non-invasive diagnosis of patients, the identified shortcomings must be addressed to optimize the process. A potential solution is the use of microfluidic devices offering high sensitivity and low production costs, making them valuable tools in cancer diagnostics [60,61]. Microfluidic systems consist of a separation zone and a detection zone [62]. The most commonly used methods for analyzing exosomes include fluorescence methods [63,64]; other electrochemical methods [65], colorimetric methods [66], and surface plasmon resonance (SPR) detection [67]. The utilization of antibody-modified magnetic beads is a common practice in fluorescence methods. An integrated microfluidic system enables the isolation and detection of plasma-derived exosomes using these magnetic beads, specifically targeting PD-L1 on the surface of the exosomes at a concentration of 10.76 per microliter in less than two hours [63]. Wu et al. developed a device for detecting exosomes in saliva as potential diagnostic indicators for head and neck squamous cell carcinoma (HNSCC) [68]. Using magnetic microspheres modified with aptamers, DNA concatemers, and quantum dots, this biosensor efficiently captures saliva exosomes and releases quantum-dot-loaded DNA concatemers for rapid one-step quantification. The technique demonstrated clinical feasibility with accuracy comparable to nanoscale flow cytometry.

Furthermore, electrochemical techniques can be integrated with microfluidic devices. An electrochemical aptasensor detects exosomes in plasma samples from breast cancer patients at various disease stages. Kashefi-Kheyrabadi et al. developed a highly sensitive and specific method for detecting cancer exosomes within 50 min, significantly reducing analysis time and sample volume compared to enzyme-linked immunosorbent assay (ELISA) [69]. For point-of-care testing, rapid and straightforward results are crucial. Colorimetric tests, offering visual responses, are particularly noteworthy [66,70]. Chen et al. developed an aptamer-modified CuS nanoparticle biosensor for the naked-eye detection of prostate cancer exosomes in urine within two hours [71]. Its non-invasive sample collection, simplicity, and low cost make it an attractive option for early prostate cancer diagnosis. Using magnetic substrates, in combination with SPR surface-enhancement Raman spectroscopy probes, demonstrated the detection of specific exosomes from breast, prostate, and colorectal cancers in blood samples [72]. The high diagnostic accuracy for detecting early-stage lung, breast, colon, liver, pancreas, and stomach cancers was achieved by analyzing surface-enhanced Raman spectroscopy profiles of exosomes using artificial intelligence. The system identified cancer presence with an AUC (Area Under Curve) of 0.970, classified the tumor organ type with a mean AUC of 0.945, achieved 90.2% sensitivity and 94.4% specificity, and correctly predicted the tumor organ in 72% of positive patients [73]. Other authors have also noted the positive benefits of combining surface-enhanced Raman spectroscopy technology in combination with artificial intelligence [74,75].

Zlotogorski-Hurvitz et al. have employed Fourier-transform infrared spectroscopy in combination with machine-learning techniques to assess the unique spectral characteristics of salivary exosomes from patients with OC and healthy individuals. This innovative approach aimed to establish a new platform for the early diagnosis of OC. The study, although involving a small number of patients (34), showed that the machine-learning model was able to discriminate salivary exosomes with a sensitivity of 100% and a specificity of 89% [76]. It is important to note that there are only a few examples in the existing literature where artificial intelligence has been used to diagnose exosomes derived from saliva.

Saliva exosomes offer a promising, non-invasive, and cost-effective tool for early cancer detection and monitoring of diseases. Advanced microfluidic devices and biosensors enhance their diagnostic potential. It is crucial to consider the diverse range of exosome isolation techniques that have a substantial impact in this field. The presented works demonstrate the employment of numerous techniques, including, but not limited to, ultracentrifugation [34,35], exclusion chromatography [46,48], precipitation [37,40], microfluidic devices [53,62], and filtration of cell culture solutions [34,43]. Despite recent advances in EV research, particularly in EV metrology and the understanding of EV biology, the field still lacks standardized parameters for comparing results, which is essential for enhancing the reliability of findings. Several challenges persist, including inconsistencies in EV nomenclature, difficulties in separating EVs from non-vesicular extracellular particles, and the need for more precise methods for EV characterization and functional analysis. Recently published comprehensive updated guidelines, “MISEV2023,” address many of these challenges by providing researchers with a comprehensive overview of current methodologies for the production, separation, and characterization of EVs from various sources, including cell cultures, body fluids, and solid tissues and saliva [33].

## 3. Role of Exosomes in Salivary Gland Tumors

The role of exosomes in the pathogenesis of SGTs extends beyond their relevance as biomarkers; they also actively participate in tumor progression by modulating the tumor microenvironment. Recently published thorough reviews emphasize the crucial role of the TME in promoting tumor progression and resistance to therapies, with tumor-cell-derived EVs transforming normal cells into tumor-associated counterparts through the transfer of molecules like RNA and proteins. EVs, particularly exosomes, mediate intercellular communication within the TME, playing a pivotal role in cancer diagnosis and therapy by targeting specific cells and facilitating their uptake via various mechanisms [77]. Evidence suggests that EVs are involved in regulating the malignant behavior of cancer cells and the formation of a premetastatic niche [78], contributing to tumor progression and metastasis [79]. Tumor-derived EVs transfer content from cancerous to non-cancerous cells, significantly affecting recipient cell behavior and creating an environment that is conducive to cancer growth, invasion, and metastasis. For instance, EVs can transfer mRNA, which translates into functional proteins and alters the proteomic profile of the target cell, while the transferred miRNAs modulate biological processes at both the transcriptional and post-transcriptional levels. Even minor changes induced by these transferred RNAs and proteins can impact normal cell metabolism [80].

Understanding the mechanisms by which cancer EVs facilitate intercellular communication and leveraging these insights for diagnostic and therapeutic applications offers significant potential for elucidating the systemic effects of cancer and enhancing patient care [81]. Xu et al. demonstrated that exosomes from salivary adenoid cystic carcinoma (SACC) cells influence associated fibroblasts and immune cells, promoting a tumor-supportive environment. In vitro, exosomes derived from SACC-83 cells were internalized by human periodontal ligament fibroblast (HPLF) cells, which subsequently enhanced the migratory and invasive capabilities of cancer cells. This interaction indicates that exosome-educated HPLF adopted a pro-tumorigenic phenotype [82]. This study highlights that exosome-mediated communication stimulates the secretion of proinflammatory cytokines and nerve growth factor (NGF) from HPLF cells. Blocking NGF reduced the enhanced invasion of SACC-83 cells, emphasizing the importance of the NGF-NTRK1 pathway in this process. These findings suggest that SACC-83 cell-derived exosomes educate HPLF cells toward a pro-tumorigenic phenotype via the NGF-NTRK1 pathway, suggesting that these exosomes are potential therapeutic targets for SACC.

Exosomes from tumors are increasingly recognized as regulators of antitumor immunity [83,84]. However, their influence on exosome secretion by distal organs remains underexplored. In vitro studies have shown that exosome production at the distal tumor site in pancreatic ductal adenocarcinoma (PDAC) prevents the development of salivary biomarker profiles. Salivary exosomes from PDAC-bearing mice suppress the tumor-killing capacity of natural killer (NK) cells. In contrast, salivary exosomes from mice with engineered tumors that suppressed exosome biogenesis did not inhibit NK cell cytotoxicity, highlighting the critical role of exosomes in modulating immune responses during tumor progression. This suppression occurs through the transfer of specific molecules that inhibit NK cell cytotoxicity, revealing a novel mechanism by which tumors evade immune surveillance. These findings underscore the dual role of salivary exosomes in carrying tumor biomarkers and modulating immune responses, providing new insights into the complex interactions between tumors and the immune system [85].

Exosomes are also involved in epithelial–mesenchymal transition (EMT), an important process by which cancer cells acquire invasive and metastatic capabilities, thereby increasing tumor malignancy [86]. Yang’s study on SACC revealed that the upregulated expression of the EGFR ligand epiregulin in SACC cells was associated with lung metastasis and poor prognosis [87]. Epiregulin is delivered via exosomes that are enriched in SACC cells. In an in vitro model, exosomes significantly enhanced lung metastasis and angiogenesis when introduced into immunodeficient mice, promoting a premetastatic niche by increasing vascular permeability. Although epiregulin-enriched exosomes play a pivotal role in metastasis by affecting both tumor and lung endothelial cells, they can be potential therapeutic targets for controlling lung metastasis in patients with SACC. High epiregulin levels were observed in SACC patients with large tumor sizes (≥4 cm) and higher clinical stages (stage III/IV). Epiregulin induces EMT by regulating GLI1/E-cadherin and increasing the expression of pro-angiogenic factors such as VEGFA, bFGF, and IL-8. These elevated levels are correlated with poor clinical outcomes, including a high incidence of lung metastasis and a high rate of local recurrence. Elevated expression or activation of epiregulin plays a role in the progression of other oral cancers, such as HNSCC [88,89]. Transforming Growth Factor β (TGFβ) was also found to be a key component of tumor-derived EVs (TEX) from HNSCC, promoting tumor growth by enhancing macrophage recruitment and angiogenesis in the TME. TGFβ+ TEX reprograms macrophages to a pro-angiogenic state without altering their M1/M2 phenotype. Experiments showed that TGFβ+ TEX increased macrophage infiltration, vascularization, and tumor progression, effects that were blocked by the TGFβ inhibitor mRER. Therefore, inhibiting TGFβ is crucial to suppress the pro-tumor functions of TEX in HNSCC [90].

Furthermore, TEX is crucial for cancer progression by preparing the premetastatic niche, aiding cancer spread, and regulating cancer cell dormancy through the transfer of molecular signals that influence recipient cell behavior. They help establish a pro-angiogenic and proinflammatory environment, promote cancer cell dissemination, and induce dormancy in dispersed cancer cells [91]. Hou et al. investigated the functions and mechanisms of SACC-derived exosomes in disease progression [92]. This study showed that exosomes derived from SACC-83 cells were internalized by their host cells, leading to increased migration and invasion capacity and enhanced endothelial cell permeability. Cancer exosomes downregulate the expression of cell junction-related proteins, such as claudins and ZO-1, which likely contribute to the promotion of migration, invasion, and metastasis. Claudins are a family of tetraspan membrane proteins that are essential for constructing tight junctions, maintaining cell polarity, and controlling paracellular permeability in epithelial and endothelial cells. The dysregulation of various claudin proteins in cancers influences cell proliferation, growth, metabolism, metastasis, and EMT, highlighting their significance in tumorigenesis and progression and their potential as diagnostic and therapeutic targets [93]. By transferring prometastatic signals, exosomes prepare distant sites for tumor cell colonization and effectively establish premetastatic niches. This capability underscores the critical role of exosomes in the metastatic cascade and their potential as targets for therapeutic intervention to prevent tumor spread [92].

Hou also investigated the role of exosomal microRNA-23b-3p (miR-23b-3p) in SACC progression, with a particular focus on angiogenesis and metastasis [94]. Exosomes transporting miR-23b-3p from SACC cells to surrounding stromal cells were found to enhance vascular permeability and reduce the levels of tight junction proteins in vitro, thereby enhancing angiogenesis and migration of SACC cells. In vivo experiments confirmed an increase in tumor microvasculature and a faster tumor growth rate in mice injected with exosomes loaded with cholesterol-modified miR-23b-3p. This study revealed that miR-23b-3p in SACC cell-derived exosomes promoted local vascular microleakage and angiogenesis by targeting the PTEN/AKT pathway, suggesting that it is a potential biomarker for distant metastasis. These findings indicate that miR-23b-3p in exosomes may be a novel therapeutic target for SACC. The miRNA hsa-miR-23b was also reported to be upregulated in salivary gland pleomorphic adenomas compared to the case in normal tissues [95]. Interestingly, the expression of miR-23b differed between normal breast and normal salivary gland tissues, but not between adenoid cystic carcinomas of the breast and salivary glands [96].

The activation of the Mitogen-Activated Protein Kinase/Extracellular Signal-Regulated Kinase pathway (MAPK/ERK) signaling pathway may be a key mechanism by which exosomes promote the proliferation of SACC cells. A study demonstrated that exosomes derived from SACC cells significantly affect tumor cell proliferation and alter ERK/P-ERK protein expression. Specifically, P-ERK expression was markedly upregulated in the experimental group relative to the control group (P < 0.05), suggesting that SACC-83-derived exosomes enhance tumor cell proliferation through the activation of the MAPK/ERK pathway. However, the exact mechanism by which exosomes trigger ERK activation in SACC-83 cells remains unclear and may involve miRNA regulation or other transcription factors, necessitating further investigation [97].

To sum up, the interactions of various cellular markers and proteins in SGTs could provide valuable insights into their roles in tumor biology. Understanding the biological mechanisms can pave the way for novel diagnostic and therapeutic strategies targeting these markers and their associated pathways, ultimately improving the management of SGTs [98]. Prominin-1 (CD133) is a protein typically found in the apical membranes of secretory and duct cells in major salivary glands. Its expression varies among different types of SGTs. It is widely expressed in adenoid cystic carcinoma, less so in acinic cell carcinoma and pleomorphic adenoma, and rarely in mucoepidermoid carcinoma, where it predominantly localizes at the apical membrane of tumor cells with acinar or intercalated duct cell differentiation. In most tissues, Prominin-1 is partially co-expressed with cancer markers such as carcinoembryonic antigen (CEA) and mucin 1 (MUC1). Prominin-1-positive vesicles contain CEA, MUC1, and several exosome-related proteins including CD63, flotillin-1, flotillin-2, and syntenin-1, the latter of which interacts with Prominin-1. The immunohistochemical profile of Prominin-1 in human salivary glands revealed its general expression at the apical plasma membrane of epithelial cells in intercalated ducts, both in normal glands and tumors, as well as in inflammatory diseases such as sialadenitis (SA). Biochemically, the ubiquitination of Prominin-1 and its interaction with syntenin-1 highlight new features of its intra- and intercellular trafficking [99].

These interactions are crucial for understanding the role of Prominin-1 in cancer development. For instance, Prominin-1 has been shown to play a promalignant role, particularly in exosomes (prom1-exo). Studies have demonstrated that exosomes from highly metastatic melanomas can increase the metastatic behavior of primary tumors by promoting bone marrow progenitors through the receptor tyrosine kinase MET [100]. Furthermore, Prominin-1 is a key regulator that ensures an appropriate response of stem cells to extracellular signals, which has significant implications for development, regeneration, and disease [101]. This regulatory role underscores the importance of Prominin-1 in maintaining cellular homeostasis and its potential involvement in tumorigenesis when dysregulated.

Despite the significant potential of exosomes in diagnosis and treatment, there is a paucity of studies that specifically address the role of exosomes in SGTs.

## 4. Exosomes in Clinical Applications for Oral Cancer

Exosomes hold great promise in the clinical management of oral diseases, particularly for the early diagnosis, prognosis, and treatment of oral cancers and radiotherapy-induced damage.

A recently published review highlighted the unique characteristics of exosomes, due to their nanoscale size, biocompatibility, targeting abilities, and ease of modification, which are ideal for use as biomarkers, therapeutic agents, and drug delivery vehicles, allowing precise delivery to cancer cells [102]. However, clinical application faces several challenges, including concerns over their in vivo safety, variability in characteristics due to different source cells and processing methods, inefficient large-scale production, and potential off-target effects. Additionally, risks such as immune recognition during drug loading complicate their use. To facilitate clinical translation, research must improve the safety, scalability, standardization, and understanding of exosome-based therapies to maximize their therapeutic potential [103]. Additionally, larger studies are needed to validate the clinical utility of exosomal markers across diverse patient populations, as current research often relies on small, homogeneous samples [104]. Natural differences such as age, sex, and genetic background, as well as specific health conditions like diabetes or cancer, can influence exosome levels that should be considered [105,106].

An example of large studies revealing the potential of extracellular vesicles and particles (EVPs) in cancer detection analyzed the proteomic profiles of EVPs in 426 human samples from tissues, plasma, and other bodily fluids. As there is a need for better biopsy tools for cancer detection, this study identified both traditional (CD9, HSPA8) and novel (β-actin, moesin) pan-EVP markers, and proteins such as versican, tenascin C, and thrombospondin 2 that distinguish tumors from normal tissues with high sensitivity and specificity. Additionally, machine-learning analysis of plasma-derived EVPs demonstrated high accuracy in detecting cancer and identifying cancer types, highlighting EVP proteins as promising biomarkers for cancer detection and classification [107].

Clinical improvement of HNSCC management may be achieved by targeting EV-related pathways. Upregulated EV-associated genes can serve as biomarkers to predict disease progression and patient response to therapies more accurately. Furthermore, strategies aimed at inhibiting EV production or neutralizing their immunosuppressive effects could enhance the efficacy of existing treatments, such as immunotherapy, and support more personalized therapeutic approaches based on the tumor’s immune profile [108]. This approach is supported by findings that demonstrate significant upregulation of EV-related genes in HNSCC cells compared to normal tissues, as shown by gene expression data from multiple cohorts, including The Cancer Genome Atlas (n = 522) and GSE65858 (n = 250). High levels of these genes were associated with increased infiltration of immunosuppressive cells, like CD4(+) T cells, macrophages, neutrophils, and dendritic cells, while reducing critical antitumor cells such as B cells and CD8(+) T cells. These genes also correlate with immunosuppressive factors NT5E and TGFB1, which contribute to further dampening of the antitumor immune response [109].

Thus, targeting EV-mediated pathways offers a promising strategy for enhancing HNSCC treatment outcomes by overcoming immune resistance and enabling more precise, individualized patient care.

Exosome levels can change due to various biological and disease-related factors. Studies indicate that exosome levels change significantly during the treatment of oral cancers, providing valuable insights into disease progression and response to therapy. In patients with oral squamous cell carcinoma (OSCC), presurgical exosome levels decrease significantly after surgery; however, higher postsurgical exosome concentrations are observed in patients who experience relapse, suggesting that exosomal levels can serve as early indicators of recurrence and prognosis [110]. Another study found that following surgical treatment for OSCC, levels of specific exosomal proteins, such as CD63 and CAV-1, also change; lower levels of CD63-positive exosomes were associated with better survival rates, while higher levels of CAV-1-positive exosomes post-surgery were linked to inflammation and potentially poorer outcomes [111]. These findings underscore the potential of exosomes as biomarkers for monitoring treatment response and predicting outcomes in OC patients.

The assessment of clinical stage and prognosis was investigated in the context of Arginase-1 (Arg-1) protein levels in both tumors and circulation of patients with HNSCC. The study found that high Arg-1 expression in tumor tissues correlated with favorable clinicopathological features and longer recurrence-free survival (RFS), while elevated plasma Arg-1 levels were linked to poor clinical outcomes. Patients with low tumor Arg-1 expression but high plasma Arg-1 levels exhibited nodal metastases and recurrence, attributed to Arg-1-carrying exosomes detected in all HNSCC patients. High exosomal Arg-1 levels were associated with positive lymph nodes and shorter RFS, suggesting that circulating Arg-1+ exosomes could more accurately indicate metastatic disease and recurrence risk than tissue or plasma Arg-1 levels alone [112].

Interestingly exosomes offer a promising approach for treating salivary gland (SG) dysfunction by supporting regenerative strategies and addressing the limitations of existing therapies for radiotherapy-induced damage in head and neck cancer patients. SG dysfunction is a common complication following radiotherapy for head and neck cancers, primarily due to the destruction of the secretory acini, which severely impairs the glands’ natural regenerative capacity. The complex architecture of SG acini and ducts presents challenges for regeneration, making three-dimensional (3D) bioprinting technologies a promising tool for accurately replicating these epithelial units in vitro. This approach facilitates the creation of mini-organs or organoids designed to restore SG function [113].

Regenerative medicine has also opened new avenues for SG tissue repair, particularly through the use of EVs, such as exosomes derived from stem cell secretomes. In a study by Chansaenroj, magnetic 3D bioassembly (M3DB) was used to generate SG functional organoids (SGo) and human dental pulp stem cells (hDPSC). The inclusion of fibroblast growth factor 10 (FGF10) enhanced the SGo with secretory epithelial units that exhibited SG-specific functions after 11 days in culture. Exosomes isolated from these cultures were evaluated for their size and biological activity, revealing that SGo-derived exosomes significantly promoted epithelial and neuronal growth in damaged SGs, whereas exosomes from hDPSC were less effective. Proteomic analysis identified several molecular targets associated with growth downstream of FGF10, including novel targets like semaphorins, indicating that exosomes generated via M3DB have significant potential for repairing SG epithelial damage.

Unlike traditional cell-based therapies, EVs are easily extracted, quantified, and stored for long periods, offering a stable and reliable therapeutic option. In animal models, EVs have shown favorable outcomes by promoting SG tissue regeneration and functional recovery through the targeted delivery of therapeutic molecules. Advanced techniques, such as 3D bioprinting, are being utilized to create SG organoids or spheroids that not only mimic the structure of natural SG tissue but also produce EVs that enhance tissue repair, providing a promising direction for future research. Despite these advancements, clinical applications remain limited; to date, human adipose-derived mesenchymal stem cell transplantation is the only method tested in Phase 1/2 clinical trials that has demonstrated significant improvements in SG function. This underscores the need for further investigation into EV-based therapies [114]. Advances in 3D bioprinting and the use of exosomes from stem cell secretomes show great potential, but further research is needed to achieve clinical applications.

Future research in exosome-based diagnostics and therapeutics should prioritize establishing standardized methods for exosome collection and analysis, developing predictive tools that leverage exosome levels for personalized care, and conducting large-scale validation studies. These efforts are essential to confirm the clinical utility of exosomal biomarkers in oral diseases and integrate them into routine clinical practice. Addressing these challenges will enable the full potential of exosome-based approaches in personalized medicine, guiding future clinical trials toward effective implementation.

Several clinical studies have investigated the role of exosomes in the diagnosis, prognosis, and treatment monitoring of head and neck cancers. One completed study, titled “Saliva and Plasma Exosomes for Oral Leukoplakia Malignant Transformation Diagnosis and Oral Cancer Prognosis Monitoring” (NCT06469892), enrollment (n = 225), focused on detecting the expression level of miR-185 in salivary and plasma exosomes to monitor oral leukoplakia and OC, sponsored by Beijing Stomatological Hospital, Capital Medical University. Another trial, “Metformin Hydrochloride in Affecting Cytokines and Exosomes in Patients With Head and Neck Cancer” (NCT03109873), enrollment (n = 9), completed with results, explored the impact of metformin hydrochloride on cytokines and exosomes in cancers of the larynx, lip, and oral cavity, with interventions including external beam radiation therapy and placebo, sponsored by Sidney Kimmel Cancer Center at Thomas Jefferson University. A study titled “Edible Plant Exosome Ability to Prevent Oral Mucositis Associated With Chemoradiation Treatment of Head and Neck Cancer” (NCT01668849), enrollment (n = 60) also completed, evaluated the use of grape extract as a dietary supplement and other drugs to prevent oral mucositis in head and neck cancer patients, conducted by the University of Louisville. Additionally, the trial “Nivolumab and BMS-986205 in Treating Patients With Stage II-IV Squamous Cell Cancer of the Head and Neck” (NCT03854032), enrollment (n = 45), which was terminated due to toxicity, aimed to evaluate the effectiveness of nivolumab in combination with the IDO1 inhibitor BMS-986205, along with therapeutic surgery, in treating advanced squamous cell cancer of the lip, oral cavity, and pharynx. The trial sought to assess the interactions between the immune and metabolic microenvironment by analyzing changes in exosome composition in peripheral blood in relation to immune, cytokine, and metabolic alterations before, during, and after treatment. The study was sponsored by Thomas Jefferson University. Lastly, an ongoing study titled “Study to Evaluate the Safety, Preliminary Efficacy, and Pharmacokinetics of 3810” (NCT03260179), enrollment (n = 60), with unknown status focuses on the drug AL3810 in patients with advanced solid tumors or advanced/metastatic colorectal cancer. Outcome measures include analyzing exosomes in plasma to quantify the content of growth factors, such as INFγ and cMyc, which can provide insights into tumor progression, immune response, and the effectiveness of therapeutic interventions in patients with HNSCC. The study was sponsored by Haihe Biopharma Co., Ltd. (Shanghai, China).

The relatively small sample sizes in most studies suggest the need for larger trials to validate these findings and establish the clinical utility of exosome-based approaches.

## 5. Conclusions

In conclusion, exosomes derived from SGT play crucial roles in tumor biology, including modulating the tumor microenvironment, influencing immune responses, and promoting metastasis. Their potential as diagnostic and prognostic biomarkers, along with their involvement in disease progression, positions them as key elements in the future of cancer management. Ongoing research aims to elucidate the mechanisms of exosome-mediated tumorigenesis and develop therapeutic strategies targeting these vesicles to improve oncological patient outcomes.

The unique properties of exosomes, such as their ability to reflect the physiological state of tumors and facilitate intercellular communication, make them invaluable in understanding tumor biology. As profiling technologies and liquid biopsy methods advance, the precision and personalization of cancer care are expected to improve significantly. By targeting the exosome-mediated pathways that contribute to tumor progression and metastasis, more effective treatments can be developed, potentially transforming the management of SGT and enhancing patient survival and quality of life.

This review underscores the importance of continued research into the intricate roles of exosomes, paving the way for breakthroughs in cancer diagnosis and therapy. Understanding the mechanisms of exosome-mediated communication and their potential as diagnostic and therapeutic targets can significantly advance cancer treatment and patient care. Despite the significant potential of exosomes in SGT diagnosis and treatment, there is a lack of literature specifically addressing their role in these tumors. Further research in this area could lead to groundbreaking advancements, ultimately transforming the landscape of cancer management.

Advances in microfluidic devices, integrated with various detection methods such as fluorescence, electrochemical, and colorimetric assays, significantly enhance the sensitivity and efficiency of exosome isolation and detection, paving the way for rapid and precise diagnostic tools in cancer research and clinical applications.

Although exosomes have significant potential in the clinical management of oral diseases, particularly for early diagnosis, prognosis, and treatment, further research is needed to address challenges related to safety, standardization, scalability, and clinical validation to fully harness their therapeutic benefits and integrate them into routine practice. Addressing these gaps will pave the way for more personalized and effective management of SGTs and other cancers.

## 6. Future Directions

Despite progress in exosome research, key aspects such as biogenesis, cargo sorting, and factors influencing secretion remain unclear, requiring further study. Current isolation techniques lack both high-throughput and high-purity screening, necessitating optimization and innovative approaches like nanocomposites with nanomaterial-modified microfluidic channels [115]. Natural exosomes exhibit therapeutic potential in antitumor, immunomodulatory, and tissue repair applications, and can enhance drug delivery accuracy and efficacy. New techniques such as cellular nanoporation and envelope protein nanocage capture show promise for overcoming loading challenges but need extensive in vivo validation. Exosome-loaded therapeutic agents are in clinical trials, indicating a future where exosomes play a central role in targeted therapies [116].

Exosome biogenesis can be inhibited by disrupting the lipid composition essential for their formation and release. Li et al. demonstrated that the neutral sphingomyelinase inhibitor GW4869 significantly inhibits tumor growth in xenograft models by reducing the proliferation, migration, and invasion of SCC7-EV cells (mouse OSCC) and decreasing the expression of IL-17A pathway molecules [117]. Similar effects were observed in earlier studies using melanoma models, where GW4869 reduced tumor growth and improved survival in mice [118]. Datta et al. identified compounds that selectively inhibit exosome biogenesis and secretion, including tipifarnib, neticonazole, climbazole, ketoconazole, and triademenol as potent inhibitors [119]. These compounds hold potential as adjunct therapeutic strategies in advanced cancer. Tipifarnib, for instance, reduces exosome levels in prostate cancer cells by inhibiting RAB27A, Alix, and nSMase2 [120]. Also, endothelin A receptor antagonists, such as sulfisoxazole (SFX) and macitentan (MAC), inhibit exosome secretion. SFX reduces vesicle release in breast cancer cell line models [121], while MAC inhibits PD-L1 exosomal secretion, enhancing antitumor immunity [122].

Future research should aim to elucidate the mechanisms of exosome-mediated tumorigenesis, delving into the ways exosomes contribute to the tumorigenesis and metastasis of SGT. This includes identifying the specific cargo within exosomes that promotes tumor progression and understanding the signaling pathways involved. Additionally, exploring the therapeutic potential of targeting exosomes is crucial. Given their role in modulating the tumor microenvironment and promoting metastasis, targeting exosome biogenesis, secretion, and uptake presents a novel therapeutic approach. Investigating inhibitors or modulators of exosomal pathways could lead to new treatments that prevent tumor spread [123].

Understanding the role of exosomes is crucial for developing innovative cancer therapies. A promising example is a novel multimodal drug delivery system using bovine milk-derived exosomes, designed specifically for targeted therapy of OSCC. This system, called Exo@Dox–EPT1, integrates doxorubicin (Dox) with a pH-sensitive bond, along with endoperoxides (EPT1) and chlorin e6 (Ce6), to enable controlled drug release and enhanced cancer cell eradication. Within the acidic tumor microenvironment, the pH-sensitive bond releases Dox, while near-infrared (NIR) light activates Ce6 to produce plasmonic heat, boosting ROS generation from EPT1. This dual pH/light-sensitive strategy offers targeted phototherapy against OSCC. Both in vitro and in vivo studies have demonstrated the biocompatibility and effectiveness of Exo@Dox–EPT1, underscoring its potential as a promising new treatment for OSCC [124].

Developing exosome-based biomarkers is another critical area of focus. There is a need to validate exosome-derived biomarkers for early detection, prognosis, and monitoring of SGT. This involves large-scale clinical studies to confirm the utility of exosomal components in clinical settings. Further, investigating how exosomes from SGT influence immune cells, such as NK cells and fibroblasts, to create a tumor-supportive microenvironment, is imperative. Understanding this interaction could uncover new strategies to enhance the body’s antitumor immune response. Moreover, the clinical application of liquid biopsies offers a non-invasive method for real-time monitoring of tumor dynamics. Future research should focus on standardizing these techniques for routine clinical use and integrating them into current diagnostic and therapeutic workflows.

Novel diagnostics require robust validation through large-scale studies to ensure their reliability, accuracy, and clinical utility. Although biomarkers are crucial for diagnosis, prediction, and guiding treatment, their effectiveness depends on demonstrating high sensitivity, specificity, and reproducibility in diverse patient populations. Large-scale studies enable the assessment of these biomarkers across varied clinical settings, reducing false-positive and false-negative results, and enhancing decision-making in clinical practice. Moreover, they help identify biomarkers that are cost-effective and suitable for widespread clinical use, ultimately improving patient care and advancing personalized medicine [125].

Finally, evaluating the potential of combining exosome-targeted therapies with existing treatments such as surgery, radiotherapy, and chemotherapy could enhance their efficacy. Research should explore the synergistic effects of such combination therapies in treating SGT.

## Figures and Tables

**Table 1:** Overview of exosome isolation methods from various biological samples.

Isolation Methods	Application (Input Material)	Principles	Advantages	Limitations	Ref.
Ultracentrifugation (UC)	- Cell culture-conditioned medium, - Plasma, - Serum, - Saliva,	- Performed by high-speed centrifugation (>100,000× *g*); isolation time: 90–120 min. - Particle separation based on size, shape and density	- Mostly suitable for isolation from large volumes, - No chemical reagents required - Low cost	- Time-consuming, - Low sample throughput, - Lower exosomes yield, - Lower purity of pellet—contamination by other small non-vesicular particles or other proteins, - Possible damage of exosomes and loss of proteins and/or DNA during the process, - Efficiency varies with the physical properties, i.e., viscosity of the biological fluid	[34,35,36,37]
Ultracentrifugation in density gradient	- Cell culture-conditioned medium, - Plasma, - Serum, - Saliva,	- Performed by high-speed centrifugation (>100,000× *g*) in specific gradient medium, i.e., sucrose, iodixanol; isolation time: 6 h, - Particle separation based on size, shape and density in a specific gradient medium	- Higher accuracy and efficiency in separation of exosomes from particles of similar density, - Higher purity of exosomes obtained (compared to UC) - Lower risk of damage or deformation of exosomes during the process,	- Time-consuming, - Labour-intensive - Highly labour intensive, - Low sample throughput - Lower exosomes yield, - Possible contamination with separation medium	[34,38,39]
Chemical precipitation (Polymer based precipitation)	- Plasma, - Serum,	- The sample is incubated with polymers, i.e., polyethylene glycol (PEG) and further precipitated by low-speed centrifugation or filtration - The polymer forms a mesh-like polymeric web that captures exosomes of a certain size, usually between 60 and 180 nm in diameter. These are later pelleted at low centrifugal speed, - There are many exosomes precipitation kits available on the market, compatible with various body fluids.	- Higher yield of exosomes (compared to UC), - Lower risk of damage or deformation of exosomes, - No required special equipment, - Less laborious - Commercial kits available on market, suitable for various biological fluids.	- Time-consuming, - Possible contamination by nonspecific particles and/or precipitation polymer, - Cannot be used for isolation of larger EVs (extracted exosome diameter 60–180 nm) - Efficiency of isolation varies with the manufacturer, - High cost of commercial kits.	[34,37,39,40]
Ultrafiltration	- Cell culture-conditioned medium, - Plasma, - Serum - Saliva, - Urine,	- The sample is passed through membrane filters that retain particles of a certain molecular weight or size, - Exosomes are separated based on their size or molecular weight.	- Less time required than ultracentrifugation (60–90 min), - No chemical reagents required, - No required special equipment, - Possibility to use membranes with different parameters (i.e., 200 nm and 30 nm) in one isolation cycle, - Commercially available solutions combine ultrafiltration with low-speed centrifugation.	- Decrease efficiency of isolation (clogging and trapping of vesicles in the filter), - Lesser exosomes yield (attachment to filter membrane and possible damage of exosomal membrane), - Possible contamination with non-vesicular particles of the same size range.	[34,41,42,43,44]
Size exclusion chromatography	- Cell culture-conditioned medium, - Plasma, - Serum, - Urine, - Saliva,	- The sample is passed through stationary phase of porous beads, - Smaller particles (i.e., proteins) pass through the pores (eluted late). Exosomes do not enter the pores (eluted at the early stage), - Particles separated based on size.	- Obtained fraction highly pure exosome, possible minimal contamination with proteins, - No chemical reagents required, - No damage to exosomal structure and preserved biological activity (high exosomes yield), - High reproducible, - High efficiency,	- Low sample throughput, - Suitable for small volumes of concentrated samples,	[34,45,46,47,48,49]
Immuno-affinity capture	- Cell culture-conditioned medium, - Plasma, - Serum, - Saliva,	- Particles separated based on receptor-ligand interaction by binding antibodies to specific antigens (i.e., CD9, CD63, CD81) on the surface of exosomes,	- High efficiency of pure exosomes, - High reproducible, - Possible to isolate specific subpopulations of exosomes, - Various commercial platform can be used (i.e., affinity chromatography columns, immune- magnetic beads, ELISA plates, microfluidic devices or immune chips)	- High reagent and used platform cost, - Time consuming and laborious (depending on the platform used), - Possible contamination with binding antibodies, - Possible loss of exosomes and lesser efficiency (strong attachment with the binding antibodies), - Loss or damage of functional activity of exosomes (using hard elution buffer), - Required cell-free samples.	[50,51,52,53,54]
